# Response of neuroblastoma cells to RF currents as a function of the signal frequency

**DOI:** 10.1186/s12885-019-6090-6

**Published:** 2019-09-05

**Authors:** María Luisa Hernández-Bule, Enrique Medel, Clara Colastra, Raquel Roldán, Alejandro Úbeda

**Affiliations:** BEM-Research Service, Ramón y Cajal University Hospital – IRYCIS, Ctra. Colmenar Viejo km 9-100, 28034 Madrid, Spain

**Keywords:** Electric currents, NB69, Capacitive-resistive electric transfer, Electrothermal therapy, Subthermal, Cytostasis, Apoptosis

## Abstract

**Background:**

Capacitive-resistive electric transfer (CRET) is a non-invasive therapeutic strategy that applies radiofrequency electric currents within the 400–600 kHz range to tissue repair and regeneration. Previous studies by our group have shown that 48 h of intermittent exposure to a 570 kHz CRET signal at a subthermal density of 50 μA/mm^2^ causes significant changes in the expression and activation of cell cycle control proteins, leading to cycle arrest in human cancer cell cultures. The present study investigates the relevance of the signal frequency in the response of the human neuroblastoma cell line NB69 to subthermal electric treatment with four different signal frequency currents within the 350–650 kHz range.

**Methods:**

Trypan blue assay, flow cytometry, immunofluorescence and immunoblot were used to study the effects of subthermal CRET currents on cell viability, cell cycle progression and the expression of several marker proteins involved in NB69 cell death and proliferation.

**Results:**

The results reveal that among the frequencies tested, only a 448 kHz signal elicited both proapoptotic and antiproliferative, statistically significant responses. The apoptotic effect would be due, at least in part, to significant changes induced by the 448 kHz signal in the expression of p53, Bax and caspase-3. The cytostatic response was preceded by alterations in the kinetics of the cell cycle and in the expression of proteins p-ERK1/2, cyclin D1 and p27, which is consistent with a potential involvement of the EGF receptor in electrically induced changes in the ERK1/2 pathway. This receives additional support from results indicating that the proapototic and antiproliferative responses to CRET can be transiently blocked when the electric stimulus is applied in the presence of PD98059, a chemical inhibitor of the ERK1/2 pathway.

**Conclusion:**

The understanding of the mechanisms underlying the ability of slowing down cancer cell growth through electrically-induced changes in the expression of proteins involved in the control of cell proliferation and apoptosis might afford new insights in the field of oncology.

## Background

Capacitive-resistive electrical transfer (CRET) therapies apply non-invasive electrothermal treatments with radiofrequency (RF) currents in the 400–600 kHz range, aimed to induce hyperthermia in targeted tissues. Due to Joule’s effect the RF current generates a thermal increase in the tissues that is a function of a number of physical and physiological parameters, including the specific impedance of each tissue [1, 2]. Hyperthermia induced by RF and microwave signals, either modulated or not, has been successfully applied in physiotherapeutic treatments for pain relief [3] or recovery of muscle, tendon and joint tissues [4–6], as well as in oncological treatments [7–10]. In the case of RF currents used in CRET therapies, in vitro experimental evidence exists providing some evidence on their potential applicability in oncology. Indeed, it has been reported that exposure to moderate levels of hyperthermia generated by 448kHz CRET currents can potentate the action of anti-tumor agents on human tongue squamous carcinoma cells HSC-4 [11], and that the potential effectiveness of CRET in cancer treatment may be enhanced by the ability of the RF current to heat metal nanoparticles embedded in the tumor tissue [12].

Until recently it has been assumed that the therapeutic effects of CRET treatments were exclusively due to the tissular response to the hyperthermia induced by RF exposure. However, several in vitro studies focused on the investigation of potential mechanisms underlying the bioeffects of CRET currents, have revealed that subthermal doses (current density J ≤ 50 μA/mm^2^; ΔT < 0.1 °C) of RF electric signal can elicit significant responses in different human cell types. We have reported that, applied at a frequency of 448 kHz, these subthermic CRET signals induce significant changes in the proliferation of adipose-derived stem cells (ADSC) obtained from healthy volunteers, as well as in their adipogenic or chondrogenic differentiation [13–15]. These results can be interpreted as indicative that, apart from the beneficial action of the electroinduced hyperthermia at the tissular level, the cellular response to the electric signal itself could significantly contribute to the therapeutic action of CRET treatments for tissue repair and regeneration.

Similar conclusions were obtained from our results on human cancer cell response to in vitro exposure to sub-thermal CRET currents. Indeed, short and repeated stimulation with 570 kHz CRET currents at a 50 μA/mm^2^ density has proven to cause significant decrease in the proliferation rate of the human hepatocarcinoma cell line HepG2. This response being due, at least in part, to arrest in phases G1 and S of the cell cycle in a fraction of the cellular population, was mediated by electrically induced changes in the expression of cycle proteins like p53 and Bcl-2. The electrical treatment also induced significant changes in the expression of alpha-fetoprotein (AFP) and albumin, both involved in the differentiation of hepatocarcinoma cells [16–18]. The same treatment induced necrosis and cell cycle arrest in the human neuroblastoma NB69 line, resulting in cytostatic and cytotoxic effects [19]. By contrast, normal, non-proliferating peripheral blood mononuclear cell (PBMC) obtained from human volunteers were irresponsive to the subthermal treatment with 570kHz currents [19].

On the basis of the foregoing, we have proposed that the efficacy of electrothermal CRET therapies may be due to a sum or cooperation between the effects of hyperthermia and the electrically-induced cellular response [19]. If so, it is conceivable that within the relatively narrow frequency spectrum of the currents applied in CRET treatments, the thermal effects may not differ significantly. However, as regards the cellular response to the electric current, it can be postulated that, as in the case of other electric, magnetic or electromagnetic stimuli, the type of induced response could differ, depending on the specific signal frequency, even within a narrow range such as that of the CRET frequencies [20, 21]. To test this hypothesis we have analyzed the response of NB69 cells to exposure to subthermal currents within a 350–650 kHz range. The results reveal that, among the tested frequencies, only a 448 kHz signal induced statistically significant proapoptotic and antiproliferative effects. The analysis of the phenomena involved in both effects revealed that the obtained cytostatic response was potentially due to modifications in cell cycle kinetics, accompanied by changes in the expression of p-ERK1/2, cyclin D1, p27 and, perhaps, of the EGF receptor at the cell membrane domain. Significant changes in the expression of p53, Bax and caspase-3 were also found, which could be involved in the observed apoptotic response.

## Methods

### Cell culture

The neuroblastoma cell line NB69 (lot No. 03I019/2008, item No. 99072802) was purchased from the European Collection of Authenticated Cell Cultures (ECACC, Salisbury, UK). The cells were periodically tested for mycoplasma contamination (PCR) and response to chemical and physical treatments, including cytostatic agents or hyperthermia.

Cells were plated in 75 cm^2^ culture flasks containing D-MEM medium (Biowhittaker, Lonza, Verviers, Belgium) supplemented with 10% (v/v) foetal bovine serum, 1% L-glutamine and 1% penicillin-fungizone (Gibco, Invitrogen, Camarillo, CA, USA). Cells were grown in an incubator (Forma Scientific, Thermo Fisher, Waltham, MA, USA) with a 37 °C, 5% CO_2_, humidified atmosphere. Every seventh day, when confluent, the culture was passed by detaching the cells with 0.05% trypsin + 0.02% EDTA (Sigma, Saint Louis, Missouri, USA) in HBSS and seeding them in a new flask. The remaining cells were plated in 60 mm-diameter Petri dishes (Nunc, Roskilde, Denmark), at a density of 8160 cells/cm^2^. For immunofluorescence analysis, the cells were seeded on glass coverslips placed inside the Petri dishes.

### Electric treatment

The electric treatment was applied at day 4th after seeding. The exposure system has been described in detail in previous papers [13, 18, 19]. Briefly, electric current was delivered by pairs of sterile, stainless steel electrodes, designed ad hoc for in vitro stimulation, that were fitted inside the Petri dishes. For CRET exposure, the electrode pairs inserted in the experimental dishes were connected in series to a Multifrequency signal generator (INDIBA®, Barcelona, Spain) custom-engineered to supply RF electric currents within a 350 kHz – 650 kHz range. For sham-exposure, the electrode pairs placed inside all control dishes were also connected to the generator, though they remained unenergized. The stimulation pattern consisted of 5-min pulses of 350 kHz, 448 kHz, 570 kHz or 650 kHz, sine wave current administered at a subthermal density of 50 μA/mm^2^. Each pulse was followed by a non-stimulation lapse of either 0 min or 25 min (short-term treatments), or 3 h and 55 min (other treatments). Except for the short-term treatment, the described pulse-interpulse cycle was repeated along total intervals of 4, 12 or 24 h. The cultures were grown in two separate, identical CO_2_ incubators (Thermo Fisher Scientific, Waltham, MA, USA). The stimulation parameters and the atmosphere inside the incubators (temperature: 37 °C, relative humidity: 90% and CO_2_: 5%) were constantly monitored. The electromagnetic environment inside the incubators was also monitored [18].

### Trypan blue assay

The effect on cell viability of a 24-h treatment with each of the selected frequencies was assessed through Trypan Blue exclusion assay. The cells were detached from the plates using 0.05% trypsin + 0.02% EDTA, stained with 0.4% Trypan Blue (Sigma, Steinheim, Germany) diluted in PBS 1:4, and counted in a Neubauer chamber. At least three experimental replicates per frequency were conducted.

### Flow cytometry

The applied standard gating procedure has been described in detail elsewhere [16, 17, 19]. Briefly, at the end of a 24-h treatment with the 448 kHz signal the cells were trypsinized, harvested and stained with propidium iodide for 1 h at room temperature. In each experimental repeat three cell suspensions were processed per experimental condition. The relative fractions of subG0/G1 (indicative of cell death) G1, S and G2/M subpopulations were determined through DNA content quantification (FACScalibur, Becton Dickinson, Franklin Lakes, NJ, USA). CellQuest 3.2 software was used for data acquisition (20,000 events per sample) and analysis. Suitable gating strategies were applied to exclude debris and aggregates.

### Immunofluorescence

The protocol has been described in detail in previously published articles [17–19]. Briefly, the expression of p-ERK1/2, p-p38, p53, Bax and caspase-3 in samples exposed on coverslips to the 448 kHz current during 30 min, 12- and/or 24-h, was immunofluorescence determined. The cells were fixed with 4% paraformaldehyde and incubated overnight at 4 °C with rabbit polyclonal anti-p-ERK1/2 (1:500; cat n: 44-680G, Thermo Fisher, Bengaluru, India), mouse monoclonal anti-p-p38 (1:500; cat. n: #9216), mouse monoclonal anti-p53 (1:200; cat. n: #2524), rabbit polyclonal anti-caspase-3 (1:400; cat. n: #9664), the three of them from Cell Signalling (Danvers, MA, USA) and rabbit polyclonal anti-Bax (1:100; cat. n: sc-6236) from Santa Cruz Technologies (Texas, USA). Afterwards, the samples were fluorescence stained for 1 h at room temperature with Alexa Fluor® 488 goat anti-rabbit IgG (1:500; cat n: A-11034) or with Alexa Fluor® 568 goat anti-mouse (1:500; cat. n: A-11031), both from Molecular Probes (Oregon, USA) and the cell nuclei were counterstained with bisBenzimide H33258 (Sigma). In each experimental repeat 3 coverslips per experimental group were photomicrographed and analyzed. Twenty microscope fields (800 cells per field) were randomly selected per coverslip and the percents of immunoreactive cells were calculated over the total cell number, revealed by Hoechst counterstaining of the nuclei using fluorescence microscopy (Nikon Eclipse TE300; Melville, USA) and Computer-Assisted Image Analysis (Analy-SIS, GMBH, Munich, Germany). All analyses were performed in duplicate and repeated at least 3 times for each of the analyzed proteins.

### Western blotting

The protocols for electrophoresis and Western blotting have been described in detail elsewhere [16]. Briefly, p-ERK1/2, were analyzed at the end of the initial 5-min stimulation with the 448-kHz signal, and at 30 min, 4 h, 12 h or 24 h from the first exposure onset. ERK1/2 and p-EGFR were analyzed at the end of the initial 5-min stimulation and 30 min afterwards. p-JNK and p-p38 expressions were analyzed at 30 min from the initial 5-min stimulation, and cyclin D1, p53, p27 and Bax were analyzed at 12 and 24 h. After CRET- or sham-stimulation the cells were harvested in hypotonic lysis buffer (10 mM Tris-HCl, 10 mM KCl, 1 mM dithiothreitol, 1 mM EDTA, 1 mM PMFS, 10 μg/ml leupeptin, 5 μg/ml pepstatin, 100 mM NaF, 20 mM β-glycerophosphate, 20 mM sodium molybdate, 0.5% Triton X-100 and 0.1% SDS). Afterwards, the proteins were separated, transferred to nitrocellulose membranes and immunostained overnight at 4 °C for rabbit polyclonal anti-p-EGFR (1:1000; cat. n: #3777), mouse monoclonal anti-p-p38 (1:1000), anti-ERK1/2 (1:1000; cat. n: #9102), anti-p-JNK (1:1000; cat. n: #4668) and anti-p53 (1:1000), them all from Cell Signalling (Danvers, MA, USA), as well as for mouse monoclonal anti-p27 (1:300; cat. n: AHZ0452; Invitrogen, Carlsbad, CA, USA), mouse monoclonal anti-Cyclin D1 (1:500; cat. n: NCL-CYCLIN D1-GM; Novocastra, Newcastle, UK), rabbit polyclonal anti-Bax (1:500; Santa Cruz Technologies) and rabbit polyclonal anti-p-ERK1/2 (1:1000; Thermo Fisher). Mouse monoclonal anti-β-actin (1:5000; cat. n: A5441; Sigma, Rehovot, Israel) was used as loading control. The membranes were incubated for 1 h at room temperature with anti-rabbit IgG conjugated to IRdye 800 CW (1:10000; cat. n: 926–32,211; LI-COR Biosciences, Nebraska, USA) and with anti-mouse IgG conjugated to IRdye 680 LT (1:15000; cat. n: 926–68,020; LI-COR Biosciences). Then, the membranes were scanned with a LI-COR Odyssey scanner (LI-COR Biosciences). When ECL-chemiluminescence was needed, the membranes were incubated with ECL-anti-mouse IgG horseradish peroxidase-linked antibody (1: 3000; cat. n: NA931; GE Healthcare, Little Chalfont, Buckinghamshire, UK) or with ECL-anti-rabbit IgG horseradish peroxidase-linked antibody (1: 3000; cat. n: NA934; GE Healthcare). The obtained bands were densitometry analyzed (PDI Quantity One 4.5.2 software, BioRad, Munich, Germany). At least three experimental replicates were conducted per time interval and protein. The data were normalized over the load controls and sham-exposed controls.

### p-ERK1/2 inhibition

Three plates with NB69 cultures were used per each of 3 experimental groups: untreated controls (C), samples treated with the p-ERK1/2 inhibitor: 20 μM PD98059 (PD), and samples exposed to CRET in the presence of the inhibitor (PD + CRET). The cultures were seeded at a density of 8160 cells/cm^2^ and incubated for 4 days. Next, PD and PD + CRET samples received the inhibitor and PD + CRET samples were exposed to the electric treatment for 12 or 24 h. After electrical stimulation, Bax expression (immunoblot at 12 h) as well as cell cycle and apoptosis rate (flow cytometry at 24 h) were analyzed following the protocols described above.

### Statistical analysis

All experimental procedures and analyses were conducted blindly for treatment. The two-tailed unpaired Student’s t-test or ANOVA and Bonferroni’s Multiple Comparison Test was applied for data analysis, using GraphPad Prism 6.01 Software (San Diego, CA, USA). Differences between samples were considered statistically significant at *p* < 0.05.

## Results

### Changes in cell viability as a function of the signal frequency

**Fig. 1:**
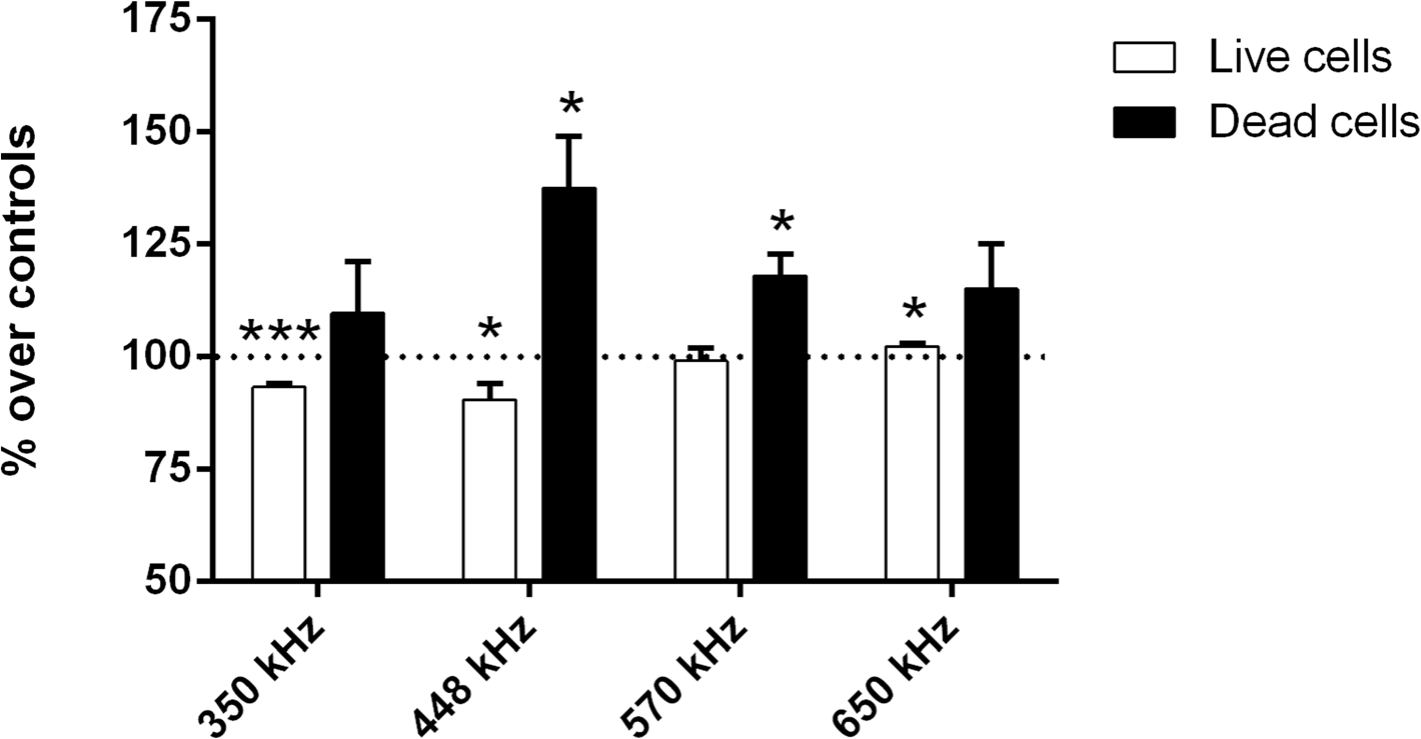
Trypan Blue assay for cell viability as a function of the signal frequency. Samples were exposed to 5-min pulses of a 50 μA/mm^2^ current density applied every 3 h 55 min, or sham-exposed, for a total of 24 h. Data are means ± SEM of at least 3 experimental repeats, normalized over the respective controls. *: 0.05 > *p* ≥ 0.01; ***: *p* < 0.001; Student’s t-test

The effects on cell death and viability after 24 h of intermittent exposure at the selected signal frequencies are summarized in Fig. 1. Compared to their respective control samples, those exposed to 350 or 448 kHz currents showed statistically significant decreases (*p* = 0.0008 and *p* = 0.0360, respectively) in the average number of living cells. By contrast, at higher frequencies the average number of living cells remained unchanged with respect to controls (*p* = 0.507 at 570 kHz) or it was slightly but significantly increased (*p* = 0.0474 at 650 kHz). Regarding cell death, the average incidence of necrosis and/or late apoptosis in the control samples was 6% of the total cell count. This death rate was significantly increased over controls in the samples exposed to 448 kHz (37%; *p* = 0.0167) and 570 kHz and (18%; *p* = 0.0109) but it did not differ from that of controls in cultures exposed to higher or lower frequency signals. Taken together, these data support the hypothesis that the cell response to electrical stimulation at sub-thermal doses is a nonlinear function of the signal frequency, being 448 kHz the most effective one in simultaneously inducing cytostatic and cytotoxic effects in NB69. On the basis of this, the 448 kHz signal was chosen as suitable to investigating the processes underlying the cytostatic/cytotoxic action of CRET sub-thermal electrostimulation.

### Effects of stimulation at 448 kHz on the cell cycle

**Fig. 2:**
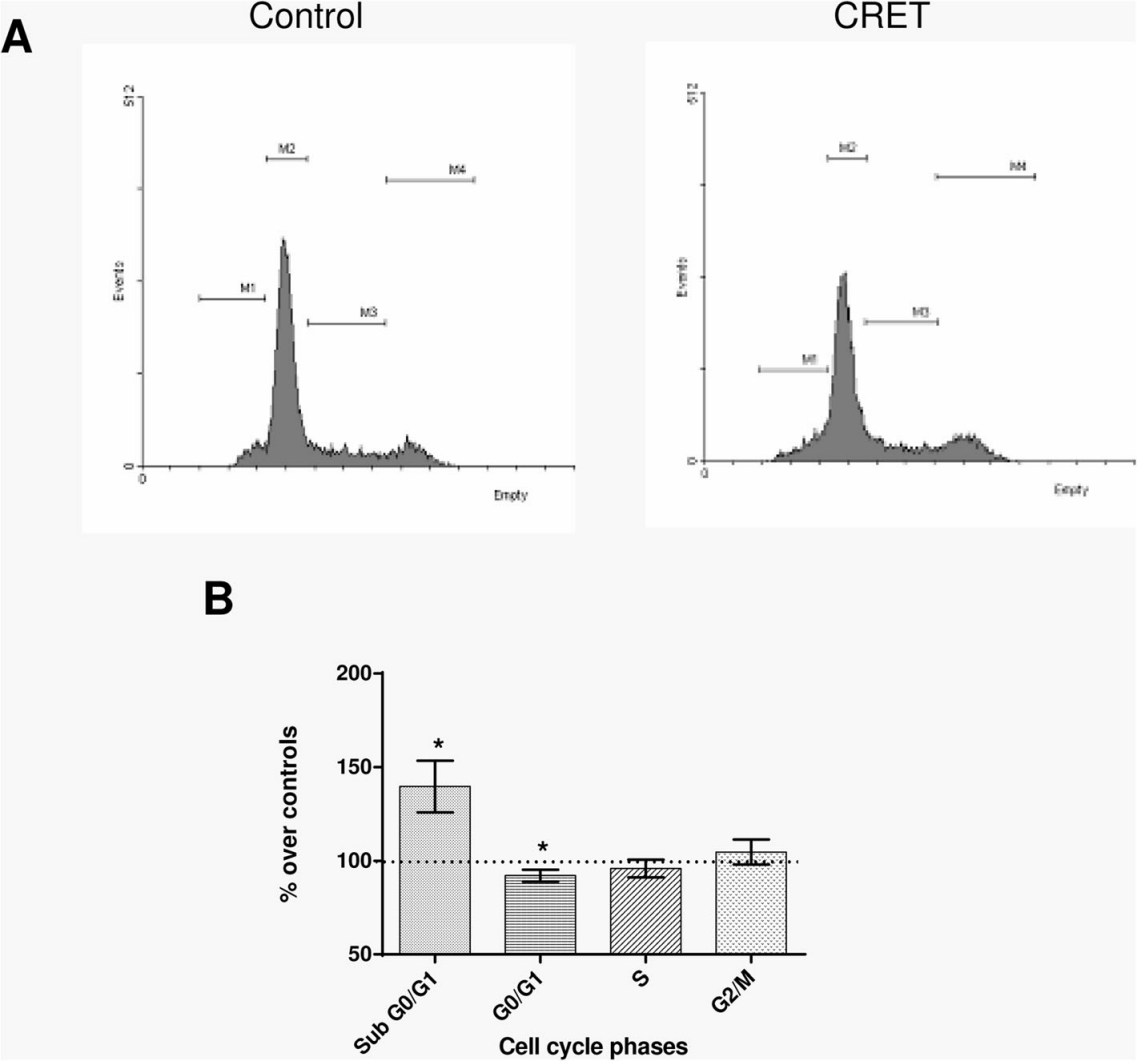
Flow cytometry analysis for cell cycle phases and apoptosis after 24 h of sham-exposure or intermittent exposure to the 448 kHz signal at a 50 μA/mm^2^ current density. **a** Representative images from one experimental run. Marker 1 (M1): region SubG0/G1; Marker 2 (M2): region G0/G1; Marker 3 (M3): region S; Marker 4 (M4): region G2/M. Events versus FL2-A parameter (PI fluorescence). **b** Percent of cells in the cell cycle phases G0/G1, S, G2/M and of apoptotic cells SubG0/G1. Data are means ± SEM of at least 3 experimental repeats, normalized over the respective sham-exposed controls. *: 0.05 > *p* ≥ 0.01; Student’s t-test

As shown in Fig. 2, flow cytometry revealed a statistically significant increase (40% over controls; *p* = 0.0136) in the percent of apoptotic nuclei, represented by the subG0/G1 peak. The electric treatment also induced slight decreases below controls in the fraction of cells in phase G1 (8%; *p* = 0.0294: statistically significant) and S (4%; *p* = 0.4075: non-significant). The possibility that these effects were mediated by sequential changes in proteins involved in apoptosis or cell cycle progression was investigated.

### Effects on proteins involved in apoptosis

**Fig. 3:**
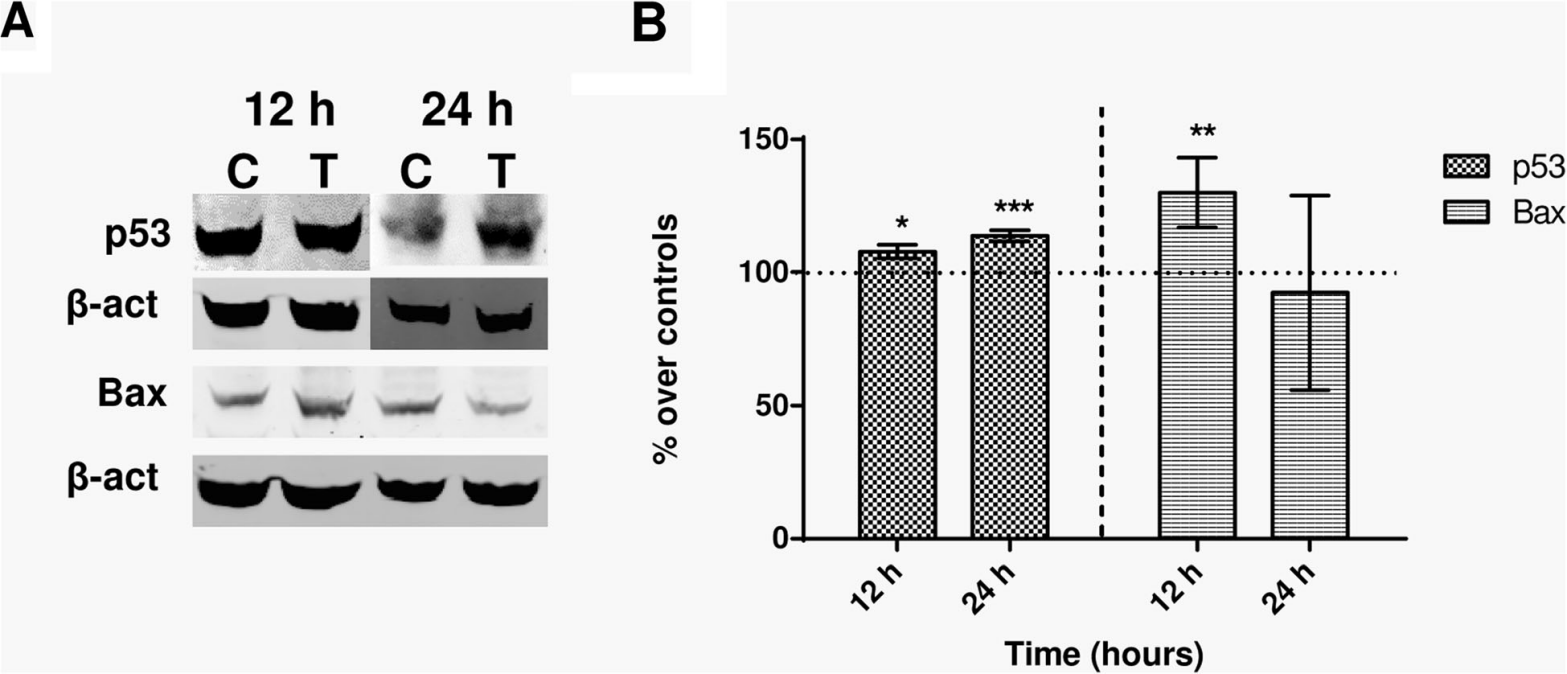
Immunoblot for p53 and Bax expression. **a** Representative blots at 12 h or 24 h of sham- or CRET-treatment. β-actin was used as loading control. 100 μg protein/lane. C = Control, T = Treated with the 448 kHz signal at 50 μA/mm^2^. **b** Immunoblot densitometry values for protein expression at 12 h or 24 h of treatment. The data, normalized over controls, are means ± SD of the protein/β-actin ratios for the corresponding proteins in at least 5 experimental repeats. *: 0.05 > *p* ≥ 0.01; **: 0.01 > *p* ≥ 0.001; ***: *p* < 0.001; Student’s t-test

**Fig. 4:**
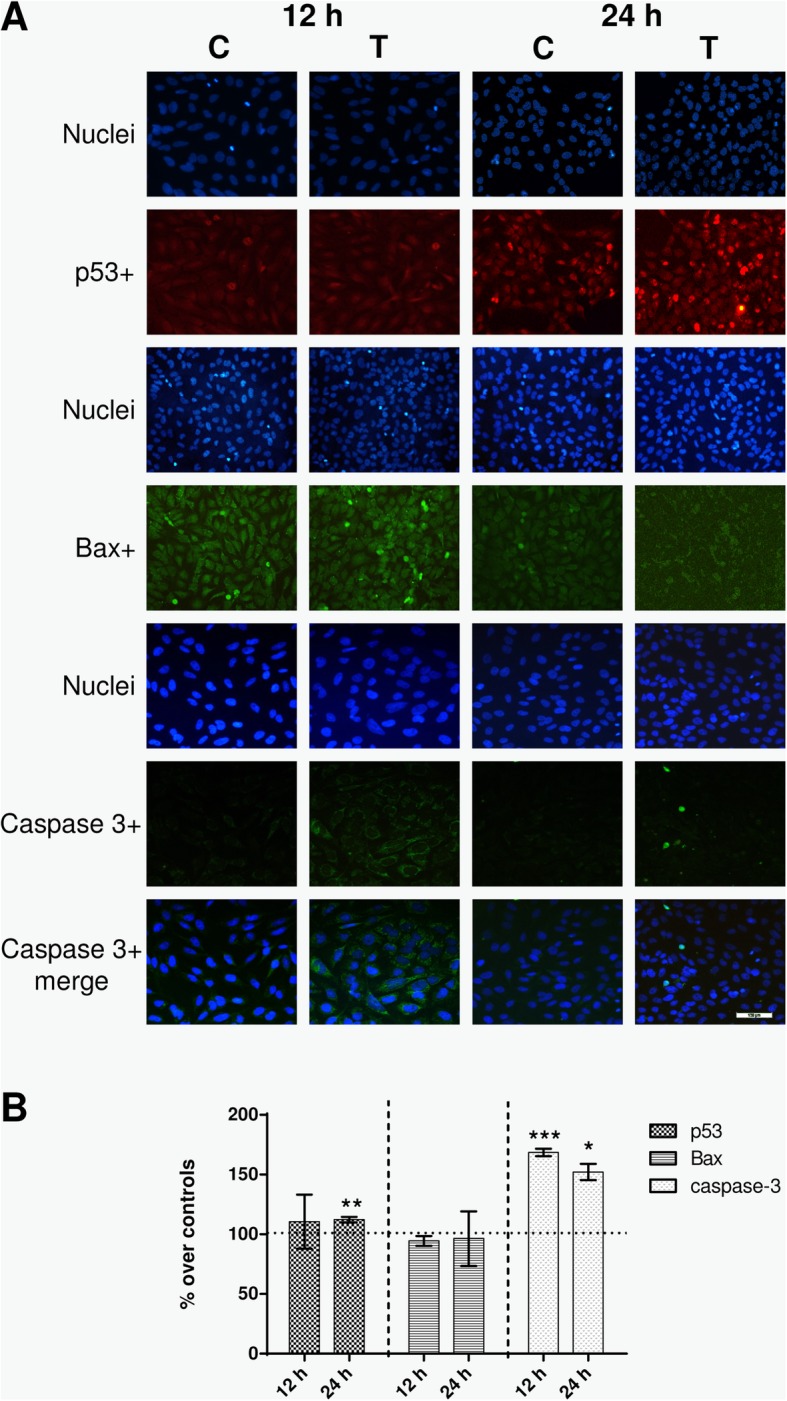
Immunofluorescence. **a** Immunofluorescence for p53, Bax and caspase-3 at 12 h or 24 h of sham- or CRET-treatment: representative micrographs. Alexa Red for p53, Alexa Green for Bax and caspase-3, and Hoechst 33258 for DNA. Bottom line displays merged images of caspase 3+ (green) and Hoechst (blue). C = Control, T = Treated with 448 kHz at 50 μA/mm^2^. Bar = 100 μm. **b** Quantification of p53, Bax and caspase-3 positive cells. Values are means ± SEM of at least 3 experimental replicates, normalized over sham-exposed controls. *: 0.05 > *p* ≥ 0.01; **: 0.01 > *p* ≥ 0.001; ***: *p* < 0.001; Student’s t-test

The tumour suppressor protein p53 promotes growth arrest, apoptosis and cellular senescence through transcriptional activation or repression of target genes such as Bax, Bak, Bcl or caspases [22]. The expression of p53, Bax and caspase-3 was analyzed in samples exposed intermittently to the 448 kHz signal for 12 h (4 exposure pulses of 5 min) or 24 h (8 pulses of 5 min). The results in Fig. 3 show that compared to their respective controls, the treated samples showed significantly increased expression of protein p53, both at 12 h (*p* = 0.0340) or 24 h (*p* < 0.0001) from the stimulation onset. This effect was confirmed by immunofluorescence analysis, although this test only could identify statistically significant (*p* = 0.065) differences with respect to controls after 24 h of exposure (Fig. 4). As for Bax expression, it experienced a significant (*p* = 0.0076), transient increase after 12 h of intermittent exposure and returned to levels equivalent of those in controls after 12 additional hours of treatment (Fig. 3). On the other hand, the immunofluorescence analysis did not provide relevant information on potential effects on Bax expression (Fig. 4). Regarding caspase-3 expression, the NB69 line is known to have very low rates of caspase-3-positive cells [23, 24]. Indeed, that rate was less than 3% in our control samples. Under these conditions the immunoblot analysis was inefficient to assess caspase-3 expression both in the exposed and in controls samples. However, the immunofluorescence assay did reveal significant increases in the amount of caspase-3+ cells, both at 12 h (68% over controls; *p* < 0.0001) and 24 h of treatment (52%; *p* = 0.0165; Fig. 4). It must be noted that the analysis of the images at 12 h of treatment revealed a large number of cells with cytoplasmic caspase labeling, while at 24 h the number of caspase+ cells was low, but their labeling had a nuclear location (Fig. 4a).

### Effects on proteins involved in cell proliferation and cell cycle control

**Fig. 5:**
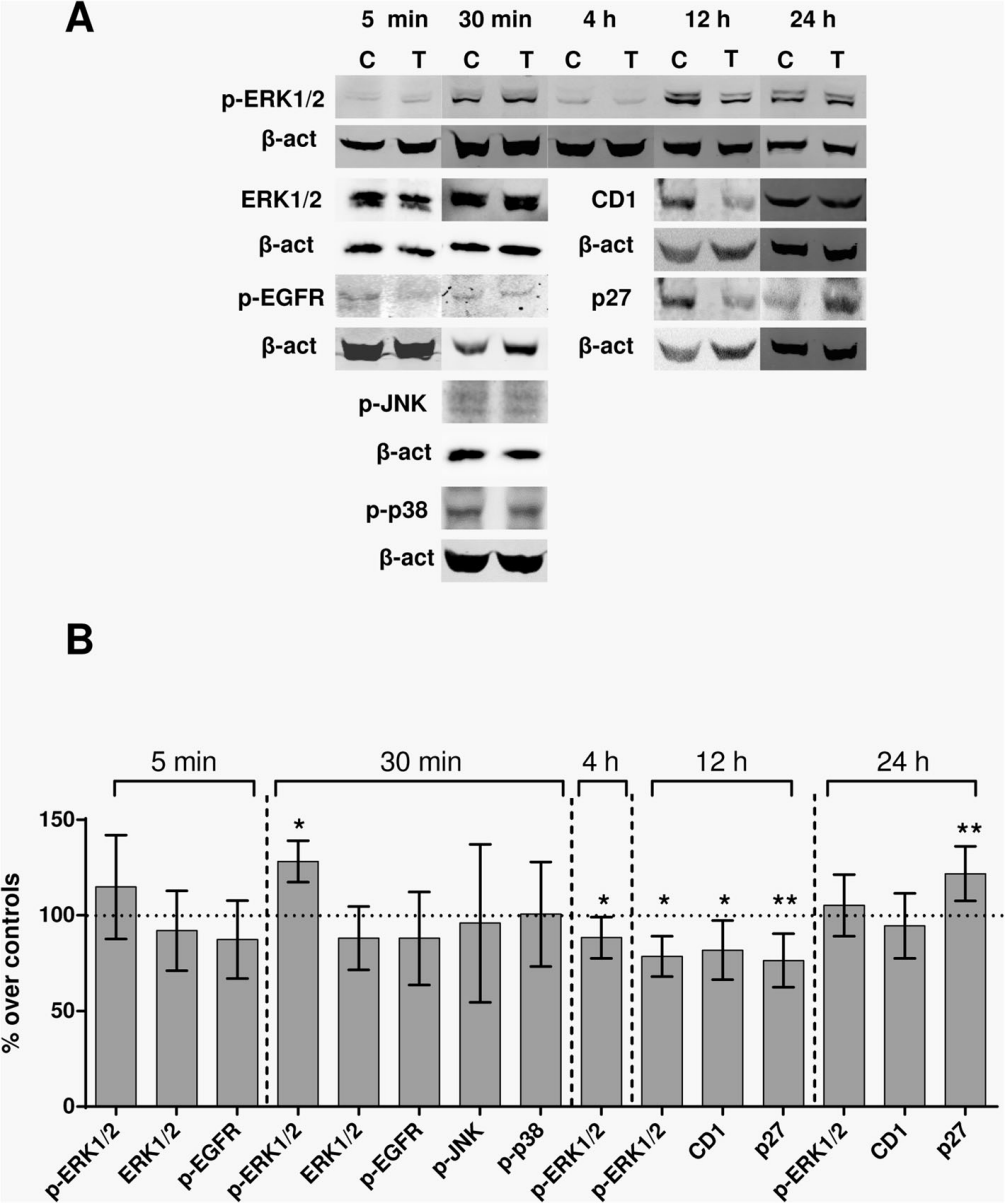
Immunoblot for p-ERK1/2, ERK1/2, p-EGFR, p-JNK, p-p38, Cyclin D1 and p27. **a** Representative blots of protein expression at 5 min, 30 min, 4 h, 12 h or 24 h of sham- or CRET- exposure to 448 kHz at 50 μA/mm^2^. 100 μg protein/lane. β-actin was used as loading control. C: sham-exposed controls, T: CRET-treated. **b** Immunoblot densitometry for protein expression. The data, normalized over controls, are means ± SD values of the protein/β-actin ratios for the corresponding proteins, at 5 min, 30 min, 4 h, 12 h or 24 h of treatment in at least 5 experimental repeats. *: 0.05 > *p* ≥ 0.01; **: 0.01 > *p* ≥ 0.001; Student’s t-test

The exposure effects on the kinetics of the expression of cyclin D1, p27, p-p38, p-ERK and the membrane receptor p-EGFR, which regulate cell proliferation and cell cycle, were investigated. Immunoblot analysis of the samples treated during 12 h revealed significant subexpression of the proteins p27 (23.6% below controls; *p* = 0.0019) and cyclin D1 (18.17% below controls; *p* = 0.0161), both involved in the control of G1 phase progression (Fig. 5). After 12 additional hours of intermittent exposure, such transient subexpression of proteins was followed by overexpression of p27 (21.8% over controls; *p* = 0.0093) and by return of the expression of cyclin D1 to levels that did not differ significantly (*p* = 0.4624) from those in controls.

It has been reported that cyclin D1 expression and activation in the early G1 phase is stimulated, through transcriptional and post-transcriptional mechanisms, by mitogenic signaling pathways such as Ras-ERK [25]. Thus, the possibility was investigated that protein p-ERK1/2, from the Ras-ERK pathway, mediates the electroinduced fluctuations in cyclin D1 expression observed throughout the CRET treatment (Fig. 5). The immunoblot data showed no significant changes in the expression of total ERK1/2 at 5 (*p* = 0.5405) and 30 min (*p* = 0.2797) of treatment, when compared to the corresponding controls. No changes were detected in the expression of the active form of the protein, p-ERK1/2 at the end of the initial 5-min exposure, either (*p* = 0.1453). However, significant overexpression of p-ERK1/2 (28.2% over controls; *p* = 0.0214) was observed at 30 min of treatment (the initial 5-min exposure plus 25 min without exposure), followed by underexpression (11.7% below controls; *p* = 0.143) at 4 h (after a second exposure pulse) and 12 h (21.4% below controls, after a fourth pulse; *p* = 0.0267). After 24 h of treatment (8 exposure pulses) the p-ERK1/2 expression levels did not differ significantly (*p* = 0.4489) from those in controls. In contrast to this early response of p-ERK1/2, other proteins such as MAPK p38, JNK or the membrane protein EGFR involved in reception of extracellular signals and in Ras-ERK pathway activation [26], did not experience significant changes in the expression of their active forms, p-p38, p-JNK and p-EGFR, after the initial 5 or 30 min of treatment (Fig. 5).

**Fig. 6:**
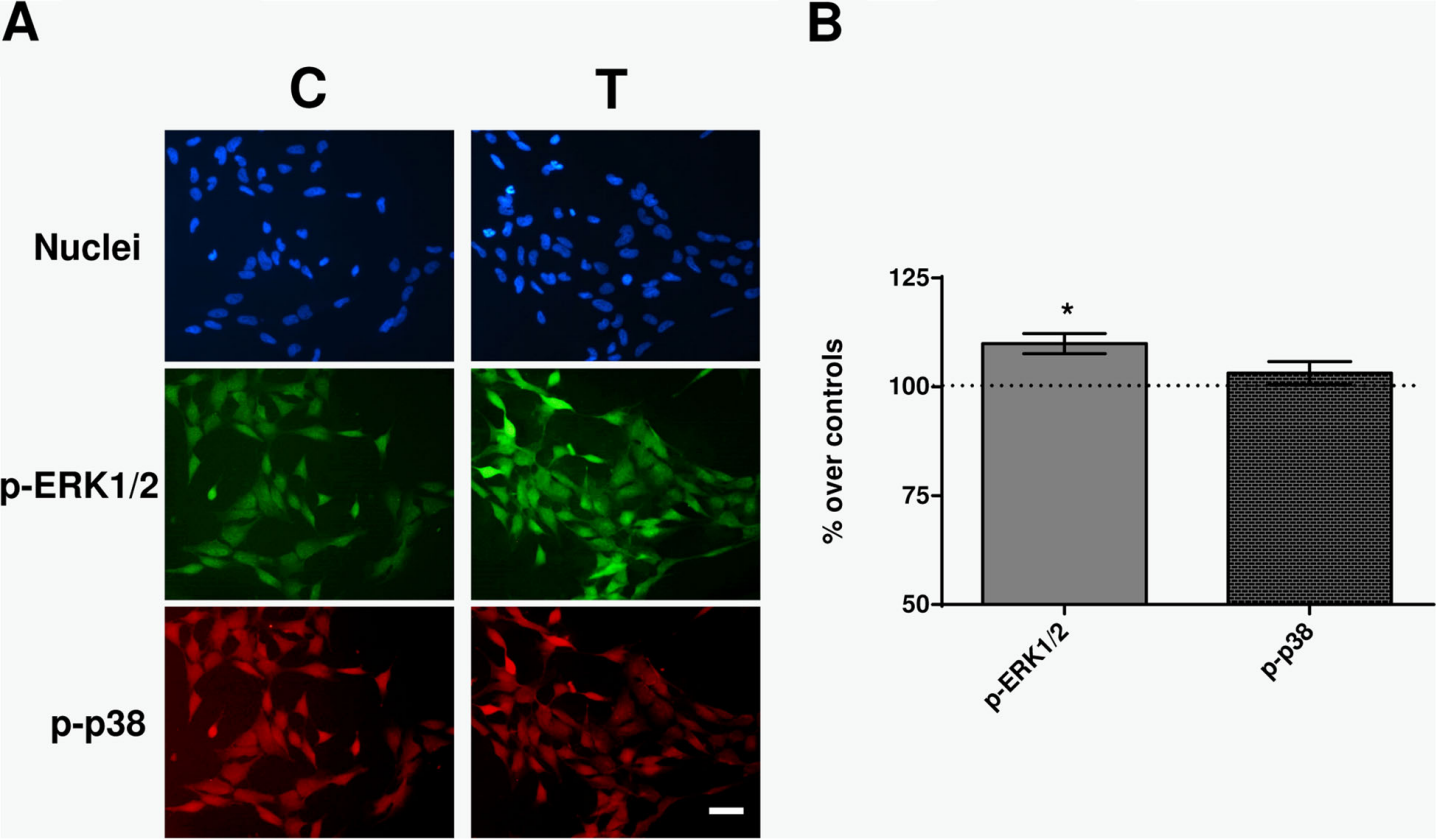
Immunofluorescence. **a** Immunofluorescence for p-ERK and p-p38 at 30 min of sham- or CRET-treatment: representative micrographs. Alexa Green for p-ERK, Alexa Red for p-p38 and Hoechst 33258 for DNA. C = Control, T = Treated with 448 kHz at 50 μA/mm^2^. Bar = 100 μm. **b** Quantification of p-ERK and p-p38 positive cells. Values are means ± SEM of 3 experimental replicates, normalized over sham-exposed controls. *: 0.05 > *p* ≥ 0.01; Student’s t-test

The CRET effects on the early expression of p-ERK and p-p38 were also analyzed by immunofluorescence. After 30 min of exposure, a significant increase (10%; *p* = 0.0261) was observed in the percent of p-ERK1/2+ cells, which in control samples averaged 60% (Fig. 6). In contrast, the average rate of p-p38+ cells at 30 min (around 50% in controls) was not changed (*p* = 0.2299) by the CRET treatment. These results were consistent with those obtained by immunoblot analysis (Fig. 5).

**Fig. 7:**
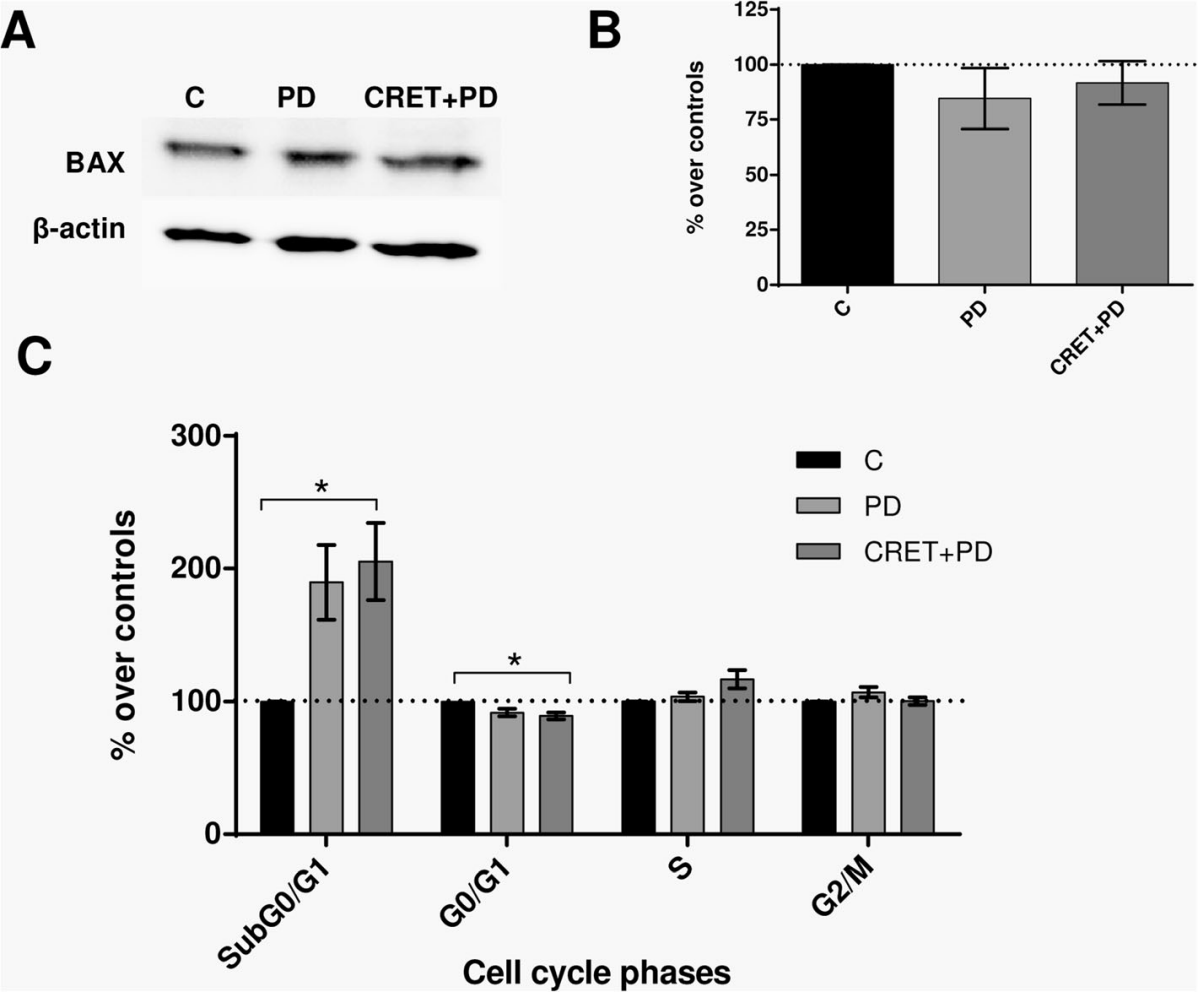
Immunoblot for Bax and flow cytometry analysis for cell cycle. **a** Representative blots of Bax at 12 h of sham-exposure (control), PD98059 inhibitor treatment, or CRET exposure in the presence of inhibitor. 100 μg protein/lane. β-actin was used as loading control. C: sham-exposed controls, PD: samples treated only with inhibitor PD98059, CRET+PD: exposed to CRET in the presence of the inhibitor. **b** Immunoblot densitometry for protein expression. The data, normalized over controls, are means ± SD values of the protein/β-actin ratios for the corresponding proteins at 12 h of treatment in 4 experimental repeats. NS: *p* ≥ 0.05; ANOVA and Bonferroni’s Multiple Comparison Test (**c**) Flow cytometry analysis for cell cycle phases and apoptosis after 24 h of sham-exposure, PD98059 treatment only or CRET-exposure in the presence of inhibitor. Percent of cells in cycle phases G0/G1, S or G2/M and of apoptotic cells SubG0/G1. Data are means ± SEM of at least 3 experimental repeats, normalized over the respective sham-exposed controls. *: 0.05 > *p* ≥ 0.01; ANOVA and Bonferroni’s Multiple Comparison Test

The possibility that the observed effects of CRET on p-ERK1/2 could influence cell cycle regulation and/or apoptosis was also investigated. The expression of Bax, which was significantly increased (30% over controls; *p* = 0.0340) after 12 h of electric treatment (Fig. 3), remained unaffected both in samples treated with the p-ERK1/2 inhibitor only and in those stimulated electrically in the presence of the inhibitor (Fig. 7a and b). By contrast, the rate of apoptosis was significantly increased (*p* = 0.0235) after 24 h of CRET exposure in the presence of the inhibitor. As for cell cycle, while after 24 h of exposure to CRET there was a modest decrease in the rate of cells in S phase (4% below controls, Fig. 2), when applied in the presence of the inhibitor the electric treatment induced a 17% increase (*p* = 0.0574) in the proportion of cells in said cycle phase and a significant decrease (*p* = 0.0213) in G0/G1 phase (Fig. 7c). None of the treatments, either applied together or separately, induced significant effects in other cycle phases.

## Discussion

The results of the signal frequency assay confirm the previously reported sensitivity of NB69 cells to CRET [19] and support the hypothesis that the signal frequency can be a critical parameter in the cellular response to RF stimulation at subthermal current densities, said response being a nonlinear function of frequency (Fig. 1). In fact, sensitivity to specific frequencies or to frequency windows is a common characteristic in the cellular response of cancer cells to weak or subthermal electric or magnetic fields and currents [27].

As the frequency scan allowed identifying the 448 kHz signal as the most effective at simultaneously inducing cytostatic and cytotoxic effects under the assayed experimental conditions, it was decided to apply this frequency in the study of phenomena involved in the cell response to subthermal CRET stimulation. To this end, it was investigated whether the decreased proportion of live cells and increased death rate induced by the 448 kHz signal could be due to blockade of cell cycle progression and/or to increased rate of apoptosis. Indeed, apoptotic responses to 100 kHz - 300 kHz electric fields [28, 29] or to 900 MHz signals [30] have been reported in cancer cells, which is in line with the increased fraction of cells in subG0/G1 phase observed here after exposure to the 448 kHz signal (Fig. 2).

The expression of p53, Bax and caspase-3 was analyzed to elucidate whether the observed proapoptotic effect could be mediated by electrically induced alterations in the expression of these proteins involved in regulation of the apoptotic process. Protein p53, a central regulator of transcription, binds to DNA, detects potential molecule damage and activates the corresponding repair mechanisms. If the damage is irreversible, p53 activates the apoptotic pathway by transcribing apoptosis effector genes [31]. After 12 or 24 h of electric stimulation, significant increases were observed in p53 expression (Figs. 3 and 4), indicating that this protein would mediate the pro-apoptotic CRET effect detected by flow cytometry. This evidence adds to that reported previously in human hepatocarcinoma cells HepG2, which showed significant changes in p53 expression and location, associated with cytostasis, after 48-h exposure to 570-kHz subthermal current [18].

Protein p53 also acts on effectors such as caspase-3 and on modulators of apoptosis such as Bax, whose overexpression has proapoptotic effects in various cell types. In response to cell death related signals, Bax migrates from its cytoplasmic location to the mitochondria, where it promotes cytochrome C release, which activates the apoptotic cascade [32]. The obtained results reveal that, after 12 h of treatment, a transient overexpression of Bax occurred (Fig. 3), together with an increased expression of caspase-3 that was maintained until 24 h of exposure (Fig. 4). This caspase-3 overexpression was followed by the apoptotic response identified by flow cytometry as an increased fraction of cells in phase subG0/G1 (Fig. 2). For caspase-3 to be active and capable of inducing apoptosis, it must translocate from the cytoplasm to the nucleus. The raw, non-normalized data, as illustrated by the images in Fig. 4a, show a large amount of caspase+ cells at 12 h of CRET treatment. However, in most of these cells the labeling has a cytoplasmic location, which indicates that said caspase is not active and, therefore, cannot induce apoptosis. In contrast, 12 h later and for reasons that are still to be elucidated, only a few cells remain caspase+, but they show nuclear labeling, indicating that have entered apoptosis. This would explain why the increase in apoptosis at 24 h is modest in absolute values, ​​though relevant and statistically significant when normalized on controls, as shown in Fig. 4b.

On the other hand, cyclin D1 forms complexes with the cyclin-dependent kinases CDK4 and CDK6, it expresses during G1 phase and regulates the G1/S transition through Rb phosphorylation [33]. The significant subexpression of cyclin D1 registered after 12 h of treatment (Fig. 5) suggests that the above described effect of CRET on the subG0/G1 phase could be mediated by electroinduced alterations in cyclin D1 expression. Taking into account that underexpression of cyclin D1 is typical of quiescent cells, the subexpression observed at 12 h of treatment might indicate that the electric stimulus would have led a sensitive fraction of the cell population to enter apoptosis or quiescence before reaching G1 phase. Such possibility was addressed by analysis of the expression of protein p27, which is an inhibitor of the cyclin-dependent kinases that control the cell cycle progression. P27 is overexpressed in quiescent cells and exerts apoptosis control and cell cycle downregulation by specifically inhibiting CDK2 activity during phase G1 [34]. The present results showed significant subexpression of p27 after 12 h of treatment, followed by significant overexpression at 24 h (Fig. 5). This overexpression indicative of quiescence could be a causal factor in the subsequent decrease in cell population observed after 24 h of treatment.

Other proteins such as ERK1/2, of the MAPK pathway, also regulate cell cycle progression, and changes in their expression and/or activation can affect the cell proliferation rate. ERK1/2 regulates the transition from phase G1 to S, so it is essential for cells to leave quiescence and progress in the cell cycle, with the consequent increase in cyclin D1 expression and activation [35, 36]. Though the type of response triggered by ERK1/2 phosphorylation varies between different cell types and is dependent on the duration and intensity of the activation [37, 38], the proliferative response is the most common one in tumor cells [39]. Even though the MAPKs pathway has been shown sensitive to intermittent exposure to electromagnetic fields in a wide spectrum of frequencies, from extremely low [40, 41] to high and ultra-high [42], the effector mechanisms of the reported responses have not yet been fully elucidated [43]. The present study investigates whether electroinduced changes in the expression of the active form of ERK1/2 could mediate the modifications in cyclin D1 and p27 expression observed in CRET exposed samples. To that end, the sequence of changes in p-ERK1/2 expression over 24 h of treatment, starting at 5 min from the exposure onset, was immunoblot analyzed. While no changes were detected in p-ERK1/2 expression at the end of the initial 5-min exposure, significant overexpression was observed 25 min after in CRET-exposed samples (Figs. 5 and 6). Subsequent exposure intervals, with two or four stimulation cycles (4 or 12 h of treatment, respectively) resulted in significant subexpression of p-ERK1/2, whereas at the end of the 24-h treatment, after 7 exposure cycles, the p-ERK1/2 levels did not differ significantly from those in controls. On the other hand, the expression of total ERK1/2 was not affected by CRET at any of the studied intervals, indicating that the electric stimulus would act on the protein activation, but not on its gene or protein expression.

**Fig. 8:**
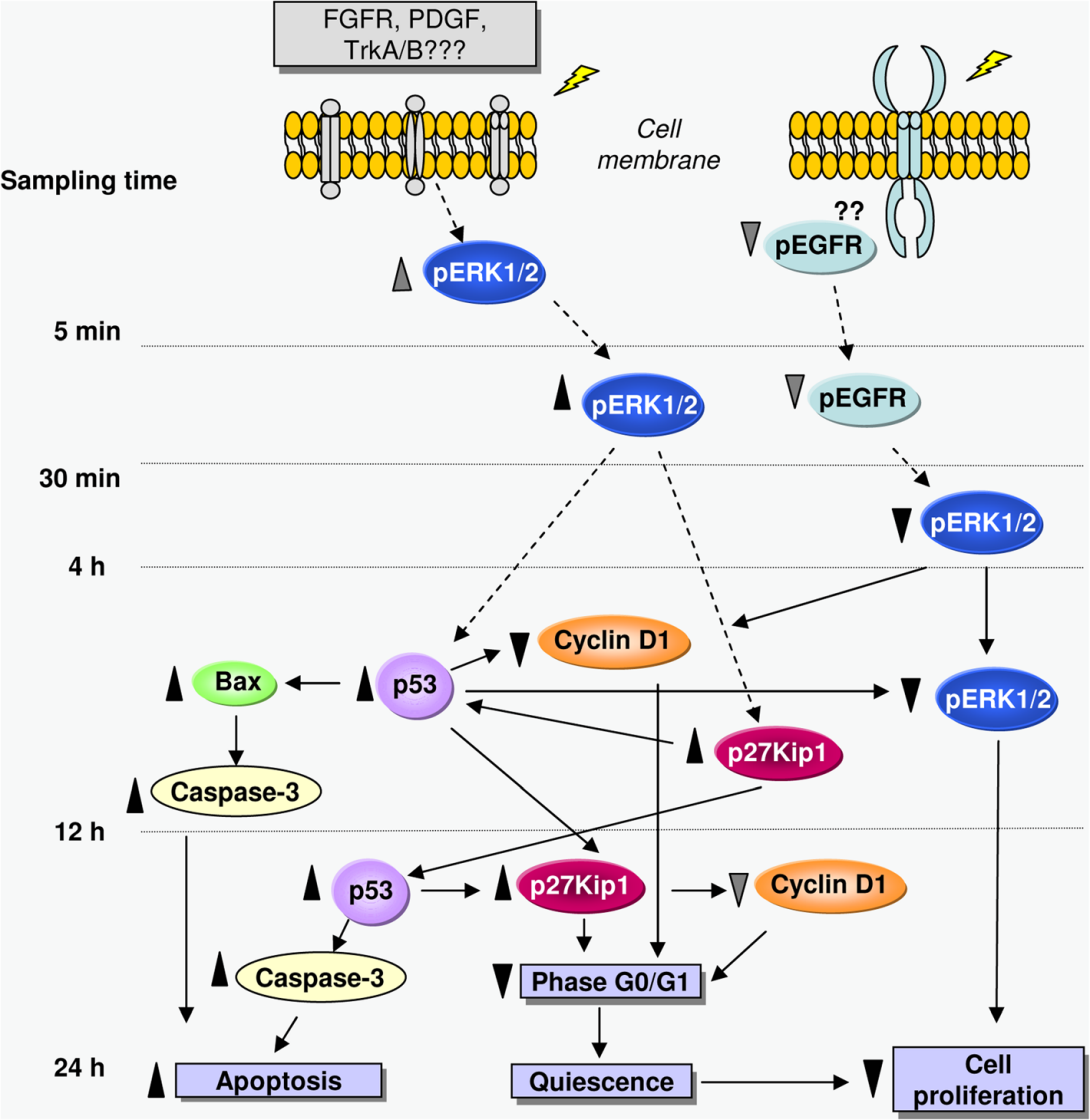
Schematic representation of the cascade of events triggered by intermittent stimulation (5 min ON/3 h and 55 min OFF) with CRET at 448 kHz and 50 μA/mm^2^. Samples were studied at 5 min, 30 min, 4 h, 12 h or 24 h after onset of the initial, 5-min exposure interval. Dotted arrows: proposed action pathways. Solid arrows: confirmed pathways; ▲: increase, statistically significant; ▼: decrease, statistically significant;: increase, not significant statistically;: decrease, not significant statistically. The antiproliferative and proapoptotic effects observed at the end of the second day of intermittent stimulation would be mediated by electrically induced changes in the expression of proteins that regulate cell cycle phases, apoptosis and cell proliferation. See the discussion text for detailed explanation

Although ERK1/2 activation plays a crucial role in promoting cell proliferation and survival, its sustained activation can also induce differentiation, senescence, cell cycle arrest and/or apoptosis in a number of cell species [39, 44]. From this, the results described above could indicate that the electric stimulation would induce an initial activation of ERK1/2 that would sustain during the first hours of treatment. Then, the continuation of this activation along ulterior stimulation pulses could trigger the subsequent subexpression of p-ERK, possibly mediated by p53 activation, and lead to the antiproliferative effect observed at the end of the 24 h of treatment (see diagram in Fig. 8).

ERK1/2 is part of the Ras-ERK MAPK pathway, which is activated by several receptor-linked tyrosine kinases such as EGFR, FGFR, PDGFR or TrkA/B, which in turn are activated by extracellular ligands. The EGFR receptor, which has been found amplified in various brain tumors [45, 46] and whose inhibition exerts antiproliferative effects in human neuroblastoma cells [47], has been proposed as a potential target sensitive to electric and magnetic fields [48, 49]. On this basis and taking into account the effects of the electric stimulation on p-ERK1/2 expression, the early effects of the electric treatment on the expression of the EGFR receptor were investigated. The immunoblot assay revealed potential decreases, non-significant statistically with respect to controls, in the expression of the receptor, both at the end of the initial, 5-min stimulation pulse and 25 min after (Fig. 5). Whether or not these slight decreases may intervene in the significant subexpression of p-ERK1/2 detected at later treatment stages, triggering the inhibition of the proliferative pathway as proposed in the scheme of Fig. 8, is a question that remains to be elucidated.

The possibility that the ERK1/2 pathway of MAPK is involved in the CRET effects on apoptosis or proliferation was further investigated by using the p-ERK1/2 inhibitor PD98059. The results showed that the presence of the inhibitor during the 12 h of electric exposure blocked the pro-apoptotic effect of CRET by reversing the electrically-induced stimulation of Bax expression (Figs. 3 and 7). However, this inhibition of the apoptotic effect of CRET could be transient, as indicated by the significant increase in the subG0/G1 cell rate observed after 24 h of treatment in the presence of PD98059. In addition, the inhibitor also blocked the decrease in the S cell rate induced by CRET when administered alone. These results provide additional support to the hypothesis that the ERK1/2 pathway could be involved in the anti-proliferative and pro-apoptotic effects of CRET (Fig. 7).

As for the MAPK protein p38, it has been shown to intervene in cell cycle control by modulating the G1/S and G2/M checkpoints [50] and is potentially involved in tumor cell survival in neuroblastoma metastatic processes [51]. This, together with the fact that p38 can express and activate in response to a variety of extracellular stimuli, including electromagnetic fields [52] makes this protein an additional candidate to be precociously involved in the cellular responses triggered by the CRET stimulus. The MAPK c-Jun N-terminal kinase (JNK) could be a potential candidate as well, since it plays a key role in cell stress, regeneration and senescence signaling pathways, and its activation has been identified as a key element in the regulation of the apoptotic signal [53]. However the immunoblot and immunofluorescence tests did not reveal significant changes in the expression of p-p38 or p-JNK after short, 30-min CRET treatment (Figs. 5 and 6), indicating that these MAPK pathways might not be sensitive to the electric signal under the assayed conditions.

## Conclusions

The block of results reported here indicates that the CRET effects in NB69 would be mediated by changes electrically induced in the expression of proteins regulating cell cycle, apoptosis and proliferation, as summarized in Fig. 8. Membrane receptors such as p-EGFR, whose expression was decreased, though not significantly, after a first pulse of treatment, could be potential initial targets of the electric stimulus. An effect of this kind could mediate in the subsequent underexpression of p-ERK1/2 (at 4 and 12 h), and lead to the antiproliferative effect observed at 24 h. On the other hand, the electric signal could be detected by other receptors such as FGFR, PDGFR or TrkA/B, which would be activated or overexpressed if they interpreted the signal as a mitogenic stimulus. This could be the cause for the increase in early p-ERK1/2 activation (at 30 min), which would mediate the succeeding subexpression of p27 (12 h). From there, the periodic repetition of the electric stimulation could lead to the subsequent activation of proteins such as p53 (at 12–24 h), and to the triggering of a proapototic response mediated by increased expression of Bax (at 12 h), caspase-3 (12–24 h) and p27 (24 h). In addition, the activation or overexpression of p53 and p27 in later phases of the treatment would intervene in the observed subexpression of cyclin D1, which would result in additional antiproliferative effect by inducing part of the cell population to enter quiescence. The specific phenomena involved in the action of p53 on apoptosis or cell cycle arrest are only partially known and are a growing subject of research. Several general factors intervening in these processes include the p53 expression levels, the type of stress signal, the cell type and the cell context at the exposure time [54].

It is obvious that, in the absence of a complete dataset on the effects of electric stimuli such as those applied in this study on other cellular and animal models, as well as in humans, the present results do not constitute a sufficient basis for propounding the application in cancer patients of such electric treatments. However, the deepening of knowledge and characterization of the sequence of responses induced in human cancer cells by subthermal electric stimuli can significantly contribute to understand the effects reported in cancer patients treated with radiofrequency currents or electric and/or magnetic fields, as well as to improve the design and safety of this type of physical treatments.

## Data Availability

All data generated or analysed during this study are included in this published article. Raw data and supplementary information are available upon request.

## Abbreviations

ADSC: Adipose-derived stem cells
AFP: Alpha-fetoprotein
CDK: Cyclin dependent kinase
CRET: Capacitive-resistive electric transfer
ECACC: European collection of authenticated cell cultures
EGF: Epidermal growth factor
EGFR: Epidermal growth factor receptor
ERK: Extracellular signal–regulated kinases
FGFR: Fibroblast growth factor receptor
JNK: c-Jun N-terminal kinase
MAPK: Mitogen-activated protein kinase
PBMC: Peripheral blood mononuclear cell
PDGFR: Platelet-derived growth factor receptors
RF: Radiofrequency
TrkA/B: Tropomyosin receptor kinase A/B

## References

[1] Kotnik T, Miklavcic D (2000). Theoretical evaluation of the distributed power dissipation in biological cells exposed to electric fields. Bioelectromagnetics.

[2] Grimnes S, Martinsen ØG, Academic press (2000). Joule effect and temperature rise. Bioimpedance and bioelectricity basics.

[3] Kumaran Binoy, Watson Tim (2019). Treatment using 448 kHz capacitive resistive monopolar radiofrequency improves pain and function in patients with osteoarthritis of the knee joint: a randomised controlled trial. Physiotherapy.

[4] Tashiro Yuto, Hasegawa Satoshi, Yokota Yuki, Nishiguchi Shu, Fukutani Naoto, Shirooka Hidehiko, Tasaka Seishiro, Matsushita Tomofumi, Matsubara Keisuke, Nakayama Yasuaki, Sonoda Takuya, Tsuboyama Tadao, Aoyama Tomoki (2017). Effect of Capacitive and Resistive electric transfer on haemoglobin saturation and tissue temperature. International Journal of Hyperthermia.

[5] Yokota Yuki, Sonoda Takuya, Tashiro Yuto, Suzuki Yusuke, Kajiwara Yu, Zeidan Hala, Nakayama Yasuaki, Kawagoe Mirei, Shimoura Kanako, Tatsumi Masataka, Nakai Kengo, Nishida Yuichi, Bito Tsubasa, Yoshimi Soyoka, Aoyama Tomoki (2018). Effect of Capacitive and Resistive electric transfer on changes in muscle flexibility and lumbopelvic alignment after fatiguing exercise. Journal of Physical Therapy Science.

[6] Bito Tsubasa, Tashiro Yuto, Suzuki Yusuke, Kajiwara Yuu, Zeidan Hala, Kawagoe Mirei, Sonoda Takuya, Nakayama Yasuaki, Yokota Yuki, Shimoura Kanako, Tatsumi Masataka, Nakai Kengo, Nishida Yuichi, Yoshimi Soyoka, Tsuboyama Tadao, Aoyama Tomoki (2019). Acute effects of capacitive and resistive electric transfer (CRet) on the Achilles tendon. Electromagnetic Biology and Medicine.

[7] Ohguri Takayuki, Yahara Katsuya, Moon Seung Dae, Yamaguchi Shinsaku, Imada Hajime, Terashima Hiromi, Korogi Yukunori (2010). Deep regional hyperthermia for the whole thoracic region using 8 MHz radiofrequency-capacitive heating device: Relationship between the radiofrequency-output power and the intra-oesophageal temperature and predictive factors for a good heating in 59 patients. International Journal of Hyperthermia.

[8] Ohguri Takayuki, Imada Hajime, Yahara Katsuya, Moon Seung Dae, Yamaguchi Shinsaku, Yatera Kazuhiro, Mukae Hiroshi, Hanagiri Takeshi, Tanaka Fumihiro, Korogi Yukunori (2012). Re-irradiation plus regional hyperthermia for recurrent non-small cell lung cancer: A potential modality for inducing long-term survival in selected patients. Lung Cancer.

[9] Zimmerman Jacquelyn W., Jimenez Hugo, Pennison Michael J., Brezovich Ivan, Morgan Desiree, Mudry Albert, Costa Frederico P., Barbault Alexandre, Pasche Boris (2013). Targeted treatment of cancer with radiofrequency electromagnetic fields amplitude-modulated at tumor-specific frequencies. Chinese Journal of Cancer.

[10] Taphoorn Martin J. B., Dirven Linda, Kanner Andrew A., Lavy-Shahaf Gitit, Weinberg Uri, Taillibert Sophie, Toms Steven A., Honnorat Jerome, Chen Thomas C., Sroubek Jan, David Carlos, Idbaih Ahmed, Easaw Jacob C., Kim Chae-Yong, Bruna Jordi, Hottinger Andreas F., Kew Yvonne, Roth Patrick, Desai Rajiv, Villano John L., Kirson Eilon D., Ram Zvi, Stupp Roger (2018). Influence of Treatment With Tumor-Treating Fields on Health-Related Quality of Life of Patients With Newly Diagnosed Glioblastoma. JAMA Oncology.

[11] Kato S, Asada R, Kageyama K, Saitoh Y, Miwa N (2011). Anticancer effects of 6-o-palmitoyl-ascorbate combined with a capacitive-resistive electric transfer hyperthermic apparatus as compared with ascorbate in relation to ascorbyl radical generation. Cytotechnology.

[12] San BH, Moh SH, Kim KK (2013). Investigation of the heating properties of platinum nanoparticles under a radiofrequency current. Int J Hyperth.

[13] Hernández-Bule María Luisa, Paíno Carlos Luis, Trillo María Ángeles, Úbeda Alejandro (2014). Electric Stimulation at 448 kHz Promotes Proliferation of Human Mesenchymal Stem Cells. Cellular Physiology and Biochemistry.

[14] Hernandez-Bule ML, Martinez-Botas J, Trillo MA, Paino CL, Ubeda A (2016). Antiadipogenic effects of subthermal electric stimulation at 448 kHz on differentiating human mesenchymal stem cells. Mol Med Rep.

[15] Hernandez-Bule ML, Trillo MA, Martinez-Garcia MA, Abilahoud C, Ubeda A. Chondrogenic differentiation of adipose-derived stem cells by radiofrequency electric stimulation. J Stem Cell Res Ther. 2017. 10.4172/2157-7633.1000407.

[16] Hernandez-Bule ML, Trillo MA, Cid MA, Leal J, Ubeda A (2007). In vitro exposure to 0.57 MHz electric currents exerts cytostatic effects in HepG2 human hepatocarcinoma cells. Int J Oncol.

[17] Hernandez-Bule ML, Cid MA, Trillo MA, Leal J, Ubeda A (2010). Cytostatic response of HepG2 to 0.57 MHz electric currents mediated by changes in cell cycle control proteins. Int J Oncol.

[18] Hernández-Bule María Luisa, Trillo María Ángeles, Úbeda Alejandro (2014). Molecular Mechanisms Underlying Antiproliferative and Differentiating Responses of Hepatocarcinoma Cells to Subthermal Electric Stimulation. PLoS ONE.

[19] Hernandez-Bule ML, Roldan E, Matilla J, Trillo MA, Ubeda A. Radiofrequency currents exert cytotoxic effects in NB69 human neuroblastoma cells but not in peripheral blood mononuclear cells. Int J Oncol. 2012. 10.3892/ijo.2012.1569. 10.3892/ijo.2012.1569 PMC3583634 22843038

[20] McNamee J. P., Chauhan V. (2009). Radiofrequency Radiation and Gene/Protein Expression: A Review. Radiation Research.

[21] Taghian T., Narmoneva D. A., Kogan A. B. (2015). Modulation of cell function by electric field: a high-resolution analysis. Journal of The Royal Society Interface.

[22] Amaral JD, Xavier JM, Steer CJ, Rodrigues CM (2010). Targeting the p53 pathway of apoptosis. Curr Pharm Des.

[23] Chen L, Malcolm AJ, Wood KM, Cole M, Variend S, Cullinane C, Pearson AD, Lunec J, Tweddle DA (2007). p53 is nuclear and functional in both undifferentiated and differentiated neuroblastoma. Cell Cycle.

[24] Qi Lei, Toyoda Hidemi, Shankar Vipin, Sakurai Naoto, Amano Keishirou, Kihira Kentaro, Iwasa Tadashi, Deguchi Takao, Hori Hiroki, Azuma Eiichi, Gabazza Esteban C., Komada Yoshihiro (2013). Heterogeneity of neuroblastoma cell lines in insulin-like growth factor 1 receptor/Akt pathway-mediated cell proliferative responses. Cancer Science.

[25] Wang C, Lisanti MP, Liao DJ (2011). Reviewing once more the c-myc and Ras collaboration: converging at the cyclin D1-CDK4 complex and challenging basic concepts of cancer biology. Cell Cycle.

[26] Martinelli Erika, Morgillo Floriana, Troiani Teresa, Ciardiello Fortunato (2017). Cancer resistance to therapies against the EGFR-RAS-RAF pathway: The role of MEK. Cancer Treatment Reviews.

[27] Porat Y, Giladi M, Schneiderman RS, Blat R, Shteingauz A, Zeevi E, Munster M, Voloshin T, Kaynan N, Tal O, et al. Determining the optimal inhibitory frequency for cancerous cells using tumor treating fields (TTFields). J Vis Exp. 2017. 10.3791/55820. 10.3791/55820 PMC5607886 28518093

[28] Giladi M, Schneiderman RS, Voloshin T, Porat Y, Munster M, Blat R, Sherbo S, Bomzon Z, Urman N, Itzhaki A, et al. Mitotic spindle disruption by alternating electric fields leads to improper chromosome segregation and mitotic catastrophe in cancer cells. Sci Rep. 2015. 10.1038/srep18046. 10.1038/srep18046 PMC4676010 26658786

[29] Giladi M, Munster M, Schneiderman RS, Voloshin T, Porat Y, Blat R, Zielinska-Chomej K, Haag P, Bomzon Z, Kirson ED, et al. Tumor treating fields (TTFields) delay DNA damage repair following radiation treatment of glioma cells. Radiat Oncol. 2017. 10.1186/s13014-017-0941-6. 10.1186/s13014-017-0941-6 PMC5747183 29284495

[30] Buttiglione M, Roca L, Montemurno E, Vitiello F, Capozzi V, Cibelli G (2007). Radiofrequency radiation (900 MHz) induces Egr-1 gene expression and affects cell-cycle control in human neuroblastoma cells. J Cell Physiol.

[31] Borriello A, Roberto R, Della Ragione F, Iolascon A (2002). Proliferate and survive: cell division cycle and apoptosis in human neuroblastoma. Haematologica.

[32] Matt Sonja, Hofmann Thomas G. (2016). The DNA damage-induced cell death response: a roadmap to kill cancer cells. Cellular and Molecular Life Sciences.

[33] Qie S, Diehl JA (2016). Cyclin D1, cancer progression, and opportunities in cancer treatment. J Mol Med.

[34] Bloom J, Pagano M (2003). Deregulated degradation of the cdk inhibitor p27 and malignant transformation. Semin Cancer Biol.

[35] Meloche S, Pouyssegur J (2007). The ERK1/2 mitogen-activated protein kinase pathway as a master regulator of the G1- to S-phase transition. Oncogene.

[36] VanArsdale T., Boshoff C., Arndt K. T., Abraham R. T. (2015). Molecular Pathways: Targeting the Cyclin D-CDK4/6 Axis for Cancer Treatment. Clinical Cancer Research.

[37] Fremin C, Meloche S. From basic research to clinical development of MEK1/2 inhibitors for cancer therapy. J Hematol Oncol. 2010. 10.1186/1756-8722-3-8. 10.1186/1756-8722-3-8 PMC2830959 20149254

[38] Bromberg-White J. L., Andersen N. J., Duesbery N. S. (2012). MEK genomics in development and disease. Briefings in Functional Genomics.

[39] Shaul YD, Seger R (2007). The MEK/ERK cascade: from signaling specificity to diverse functions. Biochim Biophys Acta.

[40] Goodman Reba, Lin-Ye Avary, Geddis Matthew S., Wickramaratne Priya J., Hodge Susan E., Pantazatos Spiro P., Blank Martin, Ambron Richard T. (2009). Extremely low frequency electromagnetic fields activate the ERK cascade, increase hsp70 protein levels and promote regeneration in Planaria. International Journal of Radiation Biology.

[41] Martínez María Antonia, Úbeda Alejandro, Cid María Antonia, Trillo María Ángeles (2012). The Proliferative Response of NB69 Human Neuroblastoma Cells to a 50 Hz Magnetic Field is mediated by ERK1/2 Signaling. Cellular Physiology and Biochemistry.

[42] Friedman J, Kraus S, Hauptman Y, Schiff Y, Seger R (2007). Mechanism of short-term ERK activation by electromagnetic fields at mobile phone frequencies. Biochem J.

[43] Francisco Artacho-Cordón, Mar Salinas-Asensio, Irene Calvente, Sandra Ríos-Arrabal, Josefa León, Elisa Román-Marinetto, Nicolás Olea, Isabel Núñez (2013). Could Radiotherapy Effectiveness Be Enhanced by Electromagnetic Field Treatment?. International Journal of Molecular Sciences.

[44] Murphy LO, Blenis J (2006). MAPK signal specificity: the right place at the right time. Trends Biochem Sci.

[45] Mimeault M, Batra SK (2007). Interplay of distinct growth factors during epithelial mesenchymal transition of cancer progenitor cells and molecular targeting as novel cancer therapies. Ann Oncol.

[46] Inaba N, Fujioka K, Saito H, Kimura M, Ikeda K, Inoue Y, Ishizawa S, Manome Y (2011). Down-regulation of EGFR prolonged cell growth of glioma but did not increase the sensitivity to temozolomide. Anticancer Res.

[47] Ho R, Minturn JE, Hishiki T, Zhao H, Wang Q, Cnaan A, Maris J, Evans AE (2005). Proliferation of human neuroblastomas mediated by the epidermal growth factor receptor. Cancer Res.

[48] Ke X. Q., Sun W. J., Lu D. Q., Fu Y. T., Chiang H. (2008). 50-Hz magnetic field induces EGF-receptor clustering and activates RAS. International Journal of Radiation Biology.

[49] Park Jeong-Eun, Seo Young-Kwon, Yoon Hee-Hoon, Kim Chan-Wha, Park Jung-Keug, Jeon Songhee (2013). Electromagnetic fields induce neural differentiation of human bone marrow derived mesenchymal stem cells via ROS mediated EGFR activation. Neurochemistry International.

[50] Thornton TM, Rincon M (2009). Non-classical p38 map kinase functions: cell cycle checkpoints and survival. Int J Biol Sci.

[51] Nolo R, Herbrich S, Rao A, Zweidler-McKay P, Kannan S, Gopalakrishnan V. Targeting P-selectin blocks neuroblastoma growth. Oncotarget. 2017. 10.18632/oncotarget.21364. 10.18632/oncotarget.21364 PMC5689715 29156825

[52] Martínez María, Úbeda Alejandro, Moreno Jorge, Trillo María (2016). Power Frequency Magnetic Fields Affect the p38 MAPK-Mediated Regulation of NB69 Cell Proliferation Implication of Free Radicals. International Journal of Molecular Sciences.

[53] Yarza R, Vela S, Solas M, Ramirez MJ. c-Jun N-terminal kinase (JNK) signaling as a therapeutic target for Alzheimer's disease. Front Pharmacol. 2015. 10.3389/fphar.2015.00321. 10.3389/fphar.2015.00321 PMC4709475 26793112

[54] Bálint E, Vousden KH (2001). Activation and activities of the p53 tumour suppressor protein. Br J Cancer.

